# Prevalence and risk indicators of early childhood caries among toddlers in Caloocan City, Philippines: a cross-sectional study

**DOI:** 10.1186/s12903-024-04407-2

**Published:** 2024-05-31

**Authors:** Maritess Oliveros-Villarico, Patimaporn Pungchanchaikul, Supatra Watthanasaen, Rajda Chaichit, Waranuch Pitiphat

**Affiliations:** 1 Department of Preventive Dentistry, Faculty of Dentistry, Khon Kaen University, Khon Kaen, Thailand; 2 Subsection of Paediatric Dentistry, Department of Clinical Dental Health Sciences, College of Dentistry, University of the Philippines, Manila, Philippines; 3 Division of Paediatric Dentistry, Department of Preventive Dentistry, Faculty of Dentistry, Khon Kaen University, Khon Kaen, Thailand; 4 Sirindhorn College of Public Health Khon Kaen, Faculty of Public Health and Allied Health Sciences, Praboromarajchanok Institute, Khon Kaen, Thailand

**Keywords:** Dental caries, Early childhood caries, Cross-sectional study, Prevalence, Risk indicator, Epidemiology

## Abstract

**Background:**

Limited published data exist on early childhood caries (ECC) among children 2 years old and below. The study aimed to determine ECC prevalence and its association with socio-demographic indicators, feeding practices and oral health behaviours among children aged 2 years and below in the Philippines.

**Methods:**

A cross-sectional study observed young children aged 4–24 months in primary health centers. Each child’s caregiver was interviewed and dental examinations were done on every child by one calibrated dentist using the ICDAS. Poisson regression using robust variance estimator analysis identified significant variables associated with ECC.

**Results:**

Seven hundred three healthy children were observed at a mean age of 13.3 ± 2.4 months. ECC prevalence was 29.2% (95% confidence interval: 26.0–32.7) among toddlers who showed a mean number of 6.7 ± 3.1 erupted teeth. Multiple regression revealed that child’s age (prevalence ratio, PR = 1.07), caregiver’s educational level (PR = 1.43), continued breastfeeding (PR = 1.36), frequent eating (PR = 1.24), visible plaque observed by the caregiver (PR = 1.34) and in the oral examination (PR = 2.90) were significant to ECC prevalence.

**Conclusions:**

ECC is alarmingly prevalent in toddlers, marked by early onset and untreated decay. Prioritizing preventive measures in the first two years of life is crucial for understanding dietary impacts and promoting oral hygiene.

**Supplementary Information:**

The online version contains supplementary material available at 10.1186/s12903-024-04407-2.

## Background

Early childhood caries (ECC) is considered as one of the most prevalent diseases of childhood [1]. It is a long-standing pandemic characterized as an early-onset, aggressive form of dental caries that affected around 1.76 billion children with primary teeth worldwide [2]. While it is highly preventable, it remains mostly untreated and continues to rise especially in underprivileged sectors of society. The prevalence of untreated caries affected 9% of children in 2010 and has remained relatively unchanged for 20 years [1]. The 2018 International Association of Paediatric Dentistry (IAPD) Global Summit on ECC abstracted data from 72 worldwide studies between 1998 and 2018 that measured caries prevalence in pre‐school children. An interesting finding was that the mean caries prevalence for 1‐year‐olds was 17% which doubled to 36% at 2 years old. The 3‐, 4‐, and 5‐year‐olds’ mean caries prevalence rates were 43%, 55%, and 63%, respectively [3]. Published studies show higher prevalence of 36–85% in Asia [4] among ages 3–5 years old. Southeast Asia presents high prevalence rates that seem understated in literature presumably because of the lack of official reports and published studies. In the Philippines, the latest national oral health survey released in 2018 revealed that 85.2% of 5-year-old respondents exhibited dental caries [5]. This prevalence appears to have remained unchanged in almost a decade, based on 2011 data that reported a prevalence rate of 87.7% in the same age group [6]. These are among the limited local information on dental caries in young children with even fewer data in the toddler age group.

In a 2021 umbrella review, potential risk factors for ECC included enamel defects, high levels of mutans streptococci, the presence of dentinal caries, increased consumption of soda, daily intake of sugary snacks, and obesity. The review recommends the need to explore the potential relationship between these risk factors and ECC through longitudinal studies [7]. The current study looked into ECC prevalence and its associated factors in toddlers to provide an insight into its initial onset possibly occurring during the early dentition period. ECC usually involves an anterior caries pattern and severe caries [8], leading to a higher risk of new caries lesions in both the primary and permanent dentition, high treatment costs, loss of school days, and diminished oral health-related quality of life [9].

Although it may be expected to have regular global reports of ECC prevalence, it is good that this type of output be made available from a population that is infrequently investigated yet recognized consistently for its high caries prevalence rates. To our knowledge, it was the first study in the country which focused solely in this specific younger age group. The present study aimed to examine the association between ECC and socio-demographic indicators, feeding practices and oral health behaviours in toddlers. The hypothesis was that these factors were associated with ECC in Filipino children.

## Methods

Two institutional review bodies gave the ethics approval (the Khon Kaen University Ethics Committee for Human Research [HE612241] and the University of the Philippines Manila Research Ethics Board [UPMREB 2018–431-01]) in compliance with the World Medical Association Declaration of Helsinki. Signed informed consent forms were obtained from the primary caregivers before participating in the study.

### Design and setting of the study

The current study was part of a longitudinal prospective study which primarily aimed to evaluate the validity of risk assessment tools for caries development. From the baseline data, the prevalence rate and potential risk indicators were generated as part of the initial cross-sectional analysis.

In contrast to the last published similar study among young rural children in 2003, an urban study site was selected this time. The study sites were community health centers familiar and easily accessible to the primary investigator in the City of Caloocan, Philippines. The study area is located in the northern part of Metro Manila with a total population of 1.58 million from a 2015 census [10] with young dependents (0–14 years old) representing 31.6% of the household [11]. It ranks third in the most populous cities of the National Capital Region [12]. Its low-income residents are known to have no natural fluoride exposure, posing a threat for caries in children. As courtesy and to facilitate the study process, official permission and support from the local government unit and its city health department were obtained prior to data collection.

Community health centers have the mandate to monitor and care for pregnant residents in their jurisdiction and are responsible for the delivery of essential vaccinations to all eligible child recipients. As such, they have a registered list of residents in their areas. Of the total 45 health centers in the city, simple random sampling selected the targeted 16 health centers that would satisfy the sample size calculated. When the health centers were identified, coordination was established for scheduling of actual data collection. Through an orientation meeting, health center workers were instructed about their valuable involvement in terms of recruitment of participants and how the study was to be conducted. Baseline data collection was from April to October 2019.

### Participants and sample size

Eligible participants were healthy children aged ≤ 24 months with at least one erupted tooth and no diagnosis of any medical or special conditions at the time of baseline data collection. Medical or special conditions refer to those requiring treatment or management by a medical professional. Excluded were children from families unable to remain in the study area for 2 years. Approximately 1,500 children who met the eligibility criteria in the health centers’ vaccination lists were invited to participate in the study.

The sample size calculation for approximating caries prevalence in this population was based on a 2003 published prevalence rate of 59% which provided the closest available proportion estimate to the toddler-aged group. However, said study was among an older group of children [13]. To achieve an estimated prevalence of 59% with a margin of error of ± 5%, a sample size of 372 was required [14]. As this cross-sectional study serves as the preliminary phase of our main prospective study, which necessitates a calculated sample size of 600, additional participants beyond the prevalence sample were needed. Accordingly, we aimed for 38–40 participants from each of the randomly selected 16 health centers.

### Data collection

A common appointment was given for eligible child participants in their respective community health centers. Each child’s primary caregiver was interviewed based on a structured questionnaire. The interview used a mix of open-ended and multiple-choice questions to collect various information. Socio-demographic questions inquired about child’s age and gender, family income, and some caregiver information. Child feeding practices were about milk-feeding habits, eating frequency and diet content. Other data relating to oral health behaviour included child toothbrushing practices, fluoride exposures and dental visits. The questionnaire was a synthesis of relevant questions from 3 caries risk assessment tools namely, Caries-risk Assessment Form by the American Academy of Pediatric Dentistry, Caries Management by Risk Assessment by the California Dental Association and the computer-based Cariogram. Questions were selected based on their relevance to the validity goals of the caries risk assessment tools. We aimed to include all questions from the CRA tools, except for those requiring clinical judgment that would not be suitable for primary caregivers to answer. To avoid redundancy, we removed duplicate questions, selecting the most complete version for inclusion in the questionnaire. Examples of removed questions include: (1) "caregiver's socioeconomic and health literacy status," which appeared in two CRA tools, (2) "frequency of sweet snacks/drinks," found in all three CRA tools, and (3) "use of daily fluoride-containing toothpaste," also present in all three CRA tools. After the final group of questions were identified, the questionnaire underwent a translation process. Although Filipinos may have a good grasp of the English language, translation into the local conversational language helped understand the questions better. The process was: (1) initial translation into Filipino done by the primary investigator; (2) back-translation done by another Filipino dental healthcare professional; (3) original and back-translated versions were compared by a third person in the same field. This process underwent several rounds until a satisfactory simple and comprehensible version was finalized (See Additional file 1 and Additional file 2). Resulting questionnaire was pre-tested with 10 Filipino mothers and adjusted accordingly for improved clarity. A visual supplementary flipchart (See Additional file 3) was created to aid the interviewers in certain questions in order for the respondents to better understand those questions. For example, the flipcharts contained photos of local common foods that are considered high- or low-sugar diet, photos of regular products with or without fluoride, or locally available toothpaste brands with or without fluoride. With the aid of the flipchart, interview duration lasted from 15–20 min. A Cariogram-derived query had the diet content featured in the questionnaire with 4 potential options for the primary caregiver participants which were combined into 2 categories during analysis. In other words, the “no/low sugar” diet represented the responses “extremely good diet with caries-inducing carbohydrate on a low level” (score 0) and “appropriate diet with sugars or other caries-inducing carbohydrate on a low level” (score 1); and “high sugar” diet for the responses “diet with relatively high content of sugars or other caries-inducing carbohydrate” (score 2) and “inappropriate diet with high intake of sugars or other caries-inducing carbohydrate” (score 3).

Prior to data collection, meetings and exercises were held. There were 10 interviewers and 4 dental assistants who alternated assignments depending on availability, and a single oral examiner. No calibrations or pre-tests for validity and reproducibility of the interview questionnaire were conducted. The meetings were basically to orient on the questionnaire and data collection process. Those who were assigned as interviewers were either non-licensed dental graduates or paediatric dentistry resident trainees. The dental assistants were actually paediatric dentists who had the task of recording the ICDAS score findings on each oral examination form as verbally dictated by the oral examiner. As for the oral examiner, the process of calibration was in the form of self-assessment tests which took several rounds until consistent 100% correct results were produced.

### Clinical examination

A single oral examiner, who is a paediatric dentist, trained in the plaque and International Caries Detection and Assessment System (ICDAS) criteria and performed clinical examination on the children. After the primary caregiver’s interview, the oral examiner conducted a visual dental check-up of the child. This was done using the knee-to-knee position with the primary caregiver, aided by artificial lighting and using a mouth mirror and sterile gauzes for wiping off saliva and debris, if needed.

Oral hygiene evaluation was conducted first by scoring the labial surfaces of primary maxillary incisors according to the modified Ramfjord visible dental plaque index [15]. The following scores indicated absence of visible plaque (score 0), easily removed thin plaque visible after drying with gauze (score 1), firmly adhered thick plaque on anterior or posterior teeth, visible without drying, associated or not to thin plaque on other teeth (score 2), and firmly adhered thick plaque, on anterior and posterior teeth, visible without drying (score 3). The sum of each examined tooth was computed and then divided by the total number of teeth included.

Then tooth status assessment evaluated caries activity and severity with the ICDAS. The scoring system was as follows: sound tooth surfaces seen without compressed air (score 0), white spot or brown discoloration in enamel when viewed wet (score 2), localized enamel breakdown with no underlying dentin shadow (score 3), underlying dark shadow from dentin (score 4), distinct cavity with visible dentin (score 5), extensive distinct cavity with visible dentin (score 6) [16]. Classification for this study’s purpose did not use score 1 which identifies non-cavitated lesions after exposure to compressed air due to the possible non-availability of compressed air in the study sites. And in consideration of the child participants’ age, score 9 was used to indicate unerupted teeth. No radiographs were taken.

Duplicate examinations were done in 71 out of 703 child participants during their respective appointments in the baseline data collection period to evaluate intra-examiner reliability. A kappa value of 0.85 indicated good agreement.

After each child’s clinical examination, the findings were briefly discussed with the accompanying primary caregiver. An ‘Oral Health Advice’ printed checklist containing individualized oral hygiene instructions on tooth brushing, toothpaste use, diet, and the next dental visit was provided (See Additional file 4 and Additional file 5). A basic hygiene kit consisting of a toothbrush, toothpaste, face towel and soap was also given to each child. When dental intervention was needed, the primary caregiver was advised to consult with either the health center dentist or their family dentist.

### Statistical analysis

SPSS Statistics for Windows, version 28.0 (IBM Corp., Armonk, NY, USA) was utilized for this purpose. A child was considered to have ECC with the presence of any non-cavitated/cavitated carious lesion in any surface of at least one tooth. Bivariate analysis determined the statistical link between the characteristics of children and ECC using t-test for continuous data and Pearson chi-square test for categorical data. The measure of association deemed appropriate for the cross-sectional study of a chronic disease was the prevalence ratio (PR) [17]. Statistical modeling strategy was based on the recommendation of Hosmer, Lemeshow, and Sturdivant (2013) who suggested using a significance level as high as 0.20 or 0.25 as a screening criterion for initial variable selection [18]. Using a traditional significance level of p ≤ 0.05 often fails to identify variables that are known to be important, and a higher level may include variables that are of questionable importance at the initial stage of model development. In the present study, variables with a *P*-value < 0.20 in the bivariate analysis proceeded to undergo a multivariable analysis. This modeling strategy allows exploration of some variables which may not be significant alone but might be with the combination of other variables. After confirming the absence of multicollinearity between independent variables (correlation < 0.7), Poisson regression analysis with robust variance estimator was performed to identify risk indicators associated with ECC. Adjusted PR and 95% confidence intervals (CIs) were calculated with *P-*values < 0.05 considered statistically significant. Multicollinearity analyses were performed among the independent variables, and none was observed. The presence of multicollinearity was determined as variance inflation factor > 5 and tolerance < 0.2.

## Results

Although the targeted sample size calculated was 600, a sizable proportion of invitees heeded the call to participate. All children, including those who were ineligible for the study, were provided the opportunity to undergo their first dental check-ups and be given proper oral health advise. Consequently, a consecutive sampling of 703 children were found eligible in the screening stage during registration together with each child’s accompanying primary caregiver. Seven-hundred three children comprised the final sample with a mean age of 13.3 ± 2.4 months (± standard deviation; range 4.2 − 24.0 months). Males represented 51.2% of the child participants. Majority of the respondents (70.0%) declared an income of less than PHP10,000.00 (USD198.07) per month classifying them as poor while the rest were middle income class [19]. Eighty-four percent were being cared for by their mothers and the other caregivers were grandparents, fathers, aunts and a few hired nannies. Half of the children were still breastfeeding and almost 80% of caregivers admitted to practice on-demand night-feeding to their child. Children who owned a toothbrush made up 49.8% but 68.0% slept without brushing their teeth after a sweet meal. Fluoride-containing toothpaste was used daily by 29.3% of child participants. Majority of the interviewed primary caregivers (57.4%) admitted to seeing obvious plaque on their child’s teeth with 27.5% of the children confirmed to have visible dental plaque from the oral examination. The socio-demographic background, feeding practices and oral health behaviours of study participants are shown in Table 1.

**Table 1:** Socio-demographic indicators, child feeding practices and oral health behaviours of study participants (n = 703)

Variables	n	%
***Socio-demographics***		
**Age**		
≥ **12 months**	505	71.8
< **12 months**	198	28.2
**Gender**		
**Male**	360	51.2
**Female**	343	48.8
**Primary caregiver**		
**Mother**	589	84.1
**Not mother**	111	15.9
**Monthly family income**		
< **Php 10,000**	492	70.0
≥ **Php10,000**	211	30.0
**Caregiver’s educational level**		
**Elementary or below**	43	6.2
**High school and up**	649	93.8
***Child Feeding Practices***		
**Continued breastfeeding**		
**Yes**	348	50.1
**No**	347	49.9
**Diet content**		
**High sugar**	281	41.4
**No/low sugar**	397	58.6
**Child nurses on demand at night**		
**Yes**	557	79.6
**No**	143	20.4
***Oral Health Behaviours***		
**Child sleeps without tooth brushing after eating sweet snacks**		
**Yes**	474	68.0
**No**	223	32.0
**Child owns a toothbrush**		
**Yes**	346	49.8
**No**	349	50.2
**Daily fluoridated toothpaste use**		
**Yes**	204	29.3
**No**	492	70.7
**Brushes child’s teeth**		
**Parent**	501	78.3
**Non-parent**	139	21.7
**Caregiver observes dental plaque on child’s teeth**		
**Yes**	396	57.4
**No**	294	42.6
**Dental plaque clinical evaluation**		
**With plaque**	193	27.5
**No plaque**	510	72.5

A total of 205 children presented with dental caries, bringing about an ECC prevalence of 29.2% (95% CI: 26.0–32.7) with a mean of 0.75 ± 1.61 carious teeth. Children with ECC were slightly older (mean age 14.3 ± 2.9 months) than those who did not exhibit ECC (12.8 ± 2.1 months). Child participants were considered in the early dentition period and had a mean of 6.7 ± 3.1 erupted teeth. Affected erupted teeth at this time were mostly primary upper incisors with a mean of 0.72 ± 1.53 carious teeth. Cavitated decay (ICDAS codes 3 to 6) were evident in 49 children or 7.0% (95% CI: 5.3–9.1) with a mean of 0.14 ± 0.61 carious teeth. The toddlers had mean cavitated decayed teeth of 0.18 ± 0.70 in the 12-months-and-older group and 0.04 ± 0.25 in the younger-than-12-months group. All decayed teeth were unrestored.

Out of all the variables considered, 17 came out as having potential associations with ECC. Tables 2 and 3 generated a descriptive summary analysis of all variables of interest and show the bivariate analyses conducted to identify variables for inclusion in the multivariable analysis. In the final model (Table 4), child’s age (PR = 1.07; 95% CI:1.02–1.12) and caregiver’s low educational level (PR = 1.43; 95% CI:1.06–1.93) were significant socio-demographic variables for ECC prevalence. Child feeding habits like continued breastfeeding (PR = 1.36; 95% CI:1.11–1.68) and eating frequency of 6 times in a day (PR = 1.24; 95% CI:1.01–1.52) were associated with increased ECC prevalence. The presence of visible plaque on a child’s teeth as observed by the primary caregiver (PR = 1.34; 95% CI:1.04–1.73) and observed in the oral examination (PR = 2.90; 95% CI:2.27–3.72) were significant risk indicators for ECC.

**Table 2:** Bivariate analysis of the association between socio-demographics and child feeding practices variables and early childhood caries (ECC) among Filipino toddlers

Variables		Presence of ECC		Crude PR (95% CI)	*P-*value
		**Yes**	**No**		
***Socio-demographics***					
**Age in months**	≥ 12	171 (33.9%)	334 (66.1%)	1.97 (1.41–2.74)	< 0.001*
	< 12	34 (17.2%)	164 (82.8%)	1	
**Caregiver’s Educational level**	Elementary or below	20 (46.5%)	23 (53.5%)	1.64 (1.16–2.31)	0.015*
	High school or above	184 (28.4%)	465 (71.6%)	1	
**Family income (in Philippine pesos)**	< 10,000	148 (30.1%)	344 (69.9%)	1.11 (0.86–1.44)	0.469
	≥ 10,000	57 (27.0%)	154 (73.0%)	1	
**Caregiver’s dental status in last 3 months**	With tooth decay	116 (32.1%)	245 (67.9%)	1.21 (0.96–1.53)	0.114*
	No tooth decay	88 (26.6%)	243 (73.4%)	1	
***Child Feeding Practices***					
**Diet content**	High-sugar	93 (33.1%)	188 (66.9%)	1.23 (0.97–1.55)	0.088*
	No/Low sugar	107 (27.0%)	290 (73.0%)	1	
**Diet frequency in 1 day**	≥ 6 times	86 (34.7%)	162 (65.3%)	1.32 (1.05–1.66)	0.024*
	< 6 times	119 (26.3%)	334 (73.7%)	1	
**Breastfeeding**	Continued	118 (33.9%)	230 (66.1%)	1.38 (1.09–1.75)	0.008*
	Discontinued	85 (24.5%)	262 (75.5%)	1	
**Received dietary advice**	No	3 (42.9%)	4 (57.1%)	1.47 (0.62–3.49)	0.423
	Yes	201 (29.1%)	490 (70.9%)	1	

^*^Variables with *p-value* < 0.20 considered in multivariable analyses

*Abbreviations*: *ECC* Early childhood caries, *PR* prevalence ratio, *CI* confidence interval

**Table 3:** Bivariate analysis of the association between oral health behaviour variables and early childhood caries (ECC) among toddlers

Variables		Presence of ECC		Crude PR (95% CI)	*P-*value
		**Yes**	**No**		
***Oral Health Behaviours***					
**Child sleeps without tooth brushing**	Yes	153 (32.3%)	321 (67.7%)	1.38 (1.06–1.82)	0.016*
	No	52 (23.3%)	171 (76.7%)	1	
**Child owns a toothbrush**	Yes	121 (35.0%)	225 (65.0%)	1.47 (1.16–1.86)	0.002*
	No	83 (23.8%)	266 (76.2%)	1	
**Brushes child’s teeth**	Non-parent	31 (22.3%)	108 (77.7%)	1.41 (1.01–1.98)	0.036*
	Parent	158 (31.5%)	343 (68.5%)	1	
**Toothbrushing frequency**	≤ 1 time/day	117 (28.9%)	288 (71.1%)	0.92 (0.73–1.16)	0.551
	2–4 times/day	87 (31.3%)	191 (68.7%)	1	
**Daily fluoride toothpaste use**	Yes	81 (39.7%)	123 (60.3%)	1.59 (1.26–2.00)	< 0.001*
	No	123 (25.0%)	369 (75.0%)	1	
**In a fluoride program**	Yes	111 (32.9%)	226 (67.1%)	1.27 (1.00–1.61)	0.052*
	No	87 (26.0%)	248 (74.0%)	1	
**Dental visit**	Once/year	49 (35.8%)	88 (64.2%)	1.29 (0.99–1.67)	0.075*
	Never	155 (27.8%)	403 (72.2%)	1	
**Caregiver is aware of child’s decayed teeth**	Yes	29 (49.2%)	30 (50.8%)	1.79 (1.34–2.39)	0.001*
	No	176 (27.5%)	464 (72.5%)	1	
**Child’s gums bleed easily**	Yes	14 (43.8%)	18 (56.3%)	1.56 (1.04–2.36)	0.070*
	No	178 (28.0%)	458 (72.0%)	1	
**Child has recent dental restorations**	Yes	2 (40.0%)	3 (60.0%)	1.39 (0.47–4.08)	0.630
	No	197 (28.8%)	486 (71.2%)	1	
**Child has health problems**	Yes	30 (38.0%)	49 (62.0%)	1.35 (0.99–1.83)	0.088*
	No	175 (28.2%)	445 (71.8%)	1	
**Child takes salivary-reducing medications**	Yes	7 (33.3%)	14 (66.7%)	1.14 (0.62–2.11)	0.636
	No	190 (29.2%)	460 (70.8%)	1	
**Caregiver sees plaque on child’s teeth**	Yes	146 (36.9%)	250 (63.1%)	1.90 (1.46–2.48)	< 0.001*
	No	57 (19.4%)	237 (80.6%)	1	
**Plaque Index**	With plaque	119 (61.7%)	74 (38.3%)	3.66 (2.93–4.57)	0.001*
	No plaque	86 (16.9%)	424 (83.1%)	1	

^*^Variables with *p-value* < 0.20 considered in multivariable analyses

*Abbreviations*: *ECC* Early childhood caries, *PR* prevalence ratio, *CI* confidence interval

**Table 4:** Risk indicators associated with early childhood caries as identified by multiple Poisson regression using robust variance estimator

Variable	Adjusted PR (95% CI)	*P-*value
**Age in months**	1.07 (1.02–1.12)	0.003
**Caregiver’s educational level**		
**Elementary or below**	1.43 (1.06–1.93)	0.019
**High school or above**	1	
**Breastfeeding**		
**Continued**	1.36 (1.11–1.68)	0.004
**Discontinued**	1	
**Diet frequency in 1 day**		
≥ **6 times**	1.24 (1.01–1.52)	0.040
< **6 times**	1	
**Caregiver observes dental plaque on child’s teeth**		
**Yes**	1.34 (1.04–1.73)	0.025
**No**	1	
**Child’s plaque index**		
**With plaque**	2.90 (2.27–3.72)	< 0.001
**No plaque**	1	

*Abbreviations*: *PR* prevalence ratio, *CI* confidence interval

## Discussion

National dental caries surveys are often lacking for children under 3, but where they have been performed a higher prevalence and severity were found compared to the permanent dentition. These cavities are almost completely untreated [20]. This study confirms that dental caries is a prevalent disease even in very young children, who are just starting to have their first teeth. Nearly one-third of the toddlers in this study had ECC. The closest local comparison comes from a 2003 survey in a northern rural area, which found a 59% prevalence rate in 2-year-olds [13]. The present findings placed the prevalence of 29% in Filipino toddlers in the lower range compared to the 2022 country-wide systematic review of Indian studies (16% to 92.2% prevalence) [21]. In the 2019 Cambodian Health and National Monitoring Study, prevalence among the 3-year-old age group was at 84.9% [22]. A global meta-analysis of cross-sectional studies using the WHO criteria estimates a 48% prevalence (95%CI 42–52) of ECC in preschool children although admittedly underestimated because only cavitated lesions were included [23].

Further analysis suggested that even within this narrow age range, ECC prevalence of urban Filipino children aged 12 months or older, was expectedly higher than those in the younger group. As a child grows older, more teeth erupt and become susceptible to ECC development. A study in Thailand reported ECC prevalence in 9-month-old children at 2%, 10 times higher in 12-month-olds (22.8%) and 30 times higher in 18-month-olds (68%) [24]. Caries was found to be significantly associated with age. A longitudinal study following 9-month-old Thai babies re-examined at 12 and 18 months found an extremely high caries-affected rate even before 18 months wherein the labial surface of maxillary incisors acquired caries 6 months after initial eruption and continued to worsen over time [25]. It became apparent in a 2021 systematic review that “age” was included as a variable in a CRA tool because it was a consistent predictor of future caries risk [26]. In this study, ECC was observed in the slightly older toddler participants and age came out as a risk indicator for ECC (PR = 1.07 for one month increase in age) (Table 4).

ECC demonstrates an atypical pattern of caries attack, particularly on smooth surfaces of upper anterior teeth [27] as seen in this study wherein newly erupted upper incisors were affected. It implicates behavioral risk factors under a person’s control, such as inappropriate feeding methods and poor oral hygiene [28]. One recognized modifiable risk factor is the presence of dental plaque showing the most significant association with ECC in a study of 9–18-month-old Thai children [24]. A 2019 review also found that the presence of plaque/debris on primary teeth was significantly and independently associated with ECC and severe ECC in Canada [29]. The current study also found that visible plaque is a significant risk indicator reported by the primary caregiver (PR 1.34) or observed clinically (PR 2.90). An explanation for the higher proportion of primary caregivers reporting a greater presence of plaque in their children as compared to the clinical findings may be due to the variance in observation time i.e., caregivers reported on overall general observations whereas the examiner referred specifically to the time of examination.

Plaque-related risk indicators like daily eating frequency of at least 6 times a day (PR 1.24) and continued breastfeeding (PR 1.36) were associated with an increased caries risk. Frequently exposing teeth to fermentable carbohydrates, especially sugars, for extended periods of time generates an acidic plaque environment, which promotes enamel demineralization and initiates dental caries [30]. While it is known that infants and babies typically need to feed every 2–3 h or seven to nine times per 24 h to satisfy their nourishment needs, it is crucial that parents and caregivers are aware of both beneficial and harmful effects of this practice. This includes the knowledge that clearing the child’s mouth thoroughly after every feeding is an oral care responsibility that will reduce acidic plaque environments from fermentable carbohydrate residues of the milk-feeding. Nursing and feeding frequency will decrease as a child grows older or as nutrition requirements change. But breastfeeding is encouraged by experts as much as the child wants or until at least 2 years old [31].

Cumulative evidence suggests that breastfeeding until the age of 1 year is not associated with an increased risk of dental caries; in fact, it may even offer protection. However, prolonged breastfeeding beyond a certain age is associated with an increased caries risk [32, 33]. A birth cohort study of Thai children confirmed the caries-protective effect of full breastfeeding up to the age of one [34]. The latest systematic review, focusing on high-quality yet limited evidence from cohort studies, concluded that breastfeeding for up to 24 months does not increase the risk of caries; however, an extended duration beyond that does raise the risk [35]. An analysis of data from the US National Health and Nutrition Examination Survey (NHANES) spanning 2011–2018, involving 3,234 children aged two to five years, reported no significant relationship between breastfeeding and ECC, and breastfeeding duration was not associated with increased caries risk [36]. Despite these differences, the majority of studies suggest that prolonged breastfeeding is associated with ECC.

Prolonged breastfeeding could not be assessed fully in this study but one-half of child participants remained breastfed at the time of data collection. The prevalence of breastmilk consumption in this population has been reported as 60% in 6–11.9 month old infants and 37% of 12–23.9 month old toddlers [31]. Although nutritionists are recommending complementary feeding behaviours that support Filipino children’s breastfeeding until 2 years of age and beyond, oral hygiene measures must be ensured for this critical period.

This study, along with others, consistently identifies frequent consumption of sweetened foods, poor oral hygiene, and visible plaque as major risk factors for ECC [37]. Preventive programs promoting regular removal of biofilm from tooth surfaces with a toothbrush, fluoride toothpaste [20], and dental floss should be advocated at the earliest appropriate time. Before birth and until the eruption of the first tooth, parents should be adequately informed about child oral health. Parental anticipatory advice needs to include dental caries risk factors and age-appropriate oral hygiene practices with focus on child oral health support until the child can perform it properly on his or her own. Currently, a considerable number of parents are not aware of or unable to perform standard preventive procedures especially brushing from the eruption of the first tooth onwards [20]. A parent’s education is an influencing factor [38] that is usually included when the complex aetiology of ECC is discussed.

A recent systematic review primarily identified low level of maternal education as a risk factor for future caries development [39]. A greater caries experience was found in child participants whose “less educated” mothers introduced them to sugar before 12 months old compared to those where sugar was introduced after 24 months of age. The conclusion was to delay sugar supply in early life [40]. In addition, reducing the frequency of sugar consumption may help prevent early onset of ECC at the outset as well as divert obesity and other serious chronic illnesses later on [41]. These are the same socio-demographic indicators and oral health behaviour factor which increased ECC risk in Filipino toddlers with the addition of continued breastfeeding and high frequency eating practices. Therefore, properly educating mothers can be the goal of oral health promotion efforts in similar community settings. Appropriate oral-health practice should be established early in the child’s life rather than break the unhealthy habit later.

Few epidemiological data on ECC in younger children inform the appropriate use of healthcare programs in the Philippines. The selected study sites have been implementing fluoride treatments with oral health education for some time. The goal of the local government is to increase the proportion of “healthy oral cavity” children entering preschool with positive oral hygiene habits and practices, ultimately reducing dental caries in primary teeth [42]. As preventive programs are most effective when started early, it is critical to consider non-cavitated lesions, aside from cavitated decay. Realizing that ECC existed in toddlers with an average of only 6.7 ± 3.1 erupted teeth informs parents and decision-makers that any preventive strategies should be actively initiated way before this stage in a child’s life.

A limitation of the study is that study sites have been confined to one city. Random cluster sampling from different health centers throughout the different cities of Metro Manila would have provided a wider sampling frame to include a larger proportion of the population. Also, our data analysis did not take into account the clustered nature of data obtained from 16 health centers assuming that the effect of ignoring the correlation may not be large if the within-cluster correlation is small. Such analysis would result in unbiased estimates of the PR, although the CIs would be narrower [43]. A major limitation of cross-sectional study was lack of temporality and thus causality cannot be established. Consequently, the cross-sectional design can only differentiate risk indicators or assumed risk factors that have been identified by prevalence data. The criteria for determining risk factors will require a longitudinal study. Extended research in the same cohort is therefore recommended to confirm the risk factors for ECC increment in this population group**.**

## Conclusions

ECC affects nearly one-third of children aged 4 to 24 months in the northern part of Metro Manila, Philippines. Several factors were linked to ECC: a child’s age, low caregiver education, continued breastfeeding, frequent feeding, and plaque build-up observed by caregivers and during clinical examinations. Therefore, stakeholders should focus preventive efforts on maternal and childcare, specifically for pre-dentate children, to implement effective ECC primary preventive programs. Empowering parents, primary caregivers, and oral health decision-makers with knowledge and resources is crucial for proactive and early management of ECC.

### Supplementary Information

Supplementary Material 1.

Supplementary Material 2.

Supplementary Material 3.

Supplementary Material 4.

Supplementary Material 5.

## Data Availability

The datasets generated and/or analysed during the current study are not publicly available in compliance with the Philippine Data Privacy Act of 2012 and the National Ethical Guidelines for Health and Health-Related Research as stated in the signed informed consents but are available from the corresponding author on reasonable request.

## Abbreviations

ECC: Early Childhood Caries
IAPD: International Association of Paediatric Dentistry
ICDAS: International Caries Detection and Assessment System
PR: Prevalence Ratio
CI: Confidence Interval
CRA: Caries Risk Assessment
NHANES: National Health and Nutrition Examination Survey
