# Supplementary material for: Prevalence and risk indicators of early childhood caries among toddlers in Caloocan City, Philippines: a cross-sectional study
Source: BMC Oral Health. 2024 May 31;24:642. doi: 10.1186/s12903-024-04407-2 (PMC11141054; doi:10.1186/s12903-024-04407-2)
Supplement: Supplementary file 1 — Supplementary Material 1. [file 12903_2024_4407_MOESM1_ESM.pdf]

PERSONAL INFORMATION

ID Number: ___________________________

NAME:____

Your Birthdate: ____

CHILD'S NAME: ____

Child's Birthdate: ____

Relation to Child: ____

Cell phone: ________________________ Other phone: ________________________

Home Address: ____

____

____

Are you responsible for feeding the child? _____YES _____NO

Are you responsible for bathing/hygienic practices of the child? _____YES_____NO

Approximately how many hours, in a day or 24 hours, are you taking care of the child? _

____HOURS

Who provides the decision for the child's health care needs? ______________________

FAMILY INCOME: _____ More than Php100,000/month

_____ Php 40,001- Php100,000/month

_____ Php 10,001- Php 40,000/month

_____ Php 10,000 or less

What is your highest level of educational attainment?

_____ a. No formal education

_____ b. Elementary ________________________

_____ c. High school ________________________

_____ d. College; ___________________________

_____ e. Post-graduate______________________

DATE: ______________________ ID: ______________________

Health Center: ______________________________

Interview Questionnaire

Mother/Caregiver information			
For Interviewer: (For Question #1, refer to Personal Information Form)<br>1. [1,2] Parent/caregiver has low socioeconomic status and or low health literacy?)	*Yes*	*No*	1. Low income bracket<br>2. Middle income bracket<br>3. High income bracket
2. [2] Mother/caregiver decay-free last 3 months?	*Yes*	*No*	*Don't know*
3. [1,2] Mother/primary caregiver has had active decay in the past 12 months?	*Yes*	*No*	*Don't know*
4. [2] Mother/caregiver chews/sucks xylitol chewing gum/lozenges 2-4x daily?	*Yes*	*No*	
5. [4] As a parent/guardian, do you receive dental check-ups regularly (every year)?	*Yes*	*No*	
Child feeding practices			
6. [4] At what age did the child stop breastfeeding?	*Yes*	*No*	If *Yes*, ___years ___months
7. [3,4] How many meals and snacks does your child eat in a day?			**0-** maximum 3 meals per day<br>**1-** 4-5 meals per day<br>**2-** 6-7 meals per day<br>**3-** more than 7 meals per day
8. [1,2,4] How often do you give sweet snacks or drinks to your child between meals during a day?			1. None<br>2. Once<br>3. 2-3 times<br>4. 4-5 times<br>5. More than 5 times
For Interviewer:<br>9. [3] Diet content			**0-** Extremely 'good' diet. Sugars or other caries-inducing carbohydrates on a very low level.<br>**1-** Appropriate diet. Sugars or other caries-inducing carbohydrates on a low level.<br>**2-** Diet with relatively high content of sugars or other caries-inducing carbohydrate.<br>**3-** Inappropriate diet. High intake of sugars or other caries-inducing carbohydrate.

10. [2] Child sleeps with a bottle?<br>11. [2] or nurses on demand?	*Yes*<br>*Yes*	*No*<br>*No*	
12. [4] When the child was 12 months old, how did the child go to sleep or take a nap?			1. Nothing in the mouth<br>2. With bottle of water<br>3. With pacifier only<br>4. Nursing on mother's breast<br>5. With bottle of milk/formula/juice<br>6. With something sweet in the mouth
13. [4] Did you ever receive advices about the relationship of diet and tooth decay from a dentist or medical doctor?	*Yes*	*No*	
Oral health behavior			
14. [4] Have you ever used a milk bottle for your child immediately before sleep/nap after the first year of his/her life?			1. Never<br>2. Occasionally<br>3. Frequently<br>4. Almost every night
15. [1,4] Does your child take sweet snacks and then sleep without brushing the teeth?			1. Never<br>2. Occasionally<br>3. Frequently<br>4. Almost every night
16. [4] Do you agree with the statement: "I have the ability to withhold frequent sugar snacks from my child between meals even when they are crying for it?"			1. Strongly agree<br>2. Agree<br>3. Neutral<br>4. Disagree<br>5. Totally disagree
17. [1] Child receives optimally fluoridated water?<br>or fluoride supplements?	*Yes*<br>*Yes*	*No*<br>*No*	*Don't know*<br>*Don't know*
18. [2,4] Has your child been living in areas without water fluoridation?			1. Yes ___years old to ___years old<br>2. No<br>3. Not sure
19. [2] Child lives in a fluoridated community?<br>or takes fluoride supplements by slowly dissolving or as chewable tablets?	*Yes*<br>*Yes*	*No*<br>*No*	*Don't know*<br>*Don't know*
20. [1,2,4] Is fluoride-containing toothpaste used for brushing your child's teeth daily?	*Yes*	*No*	*Don't know*
21. [1,4] Have you or the dentists ever used for your child any fluoride-containing			1. Yes How frequent? _____<br>2. No<br>3. Don't know

products (fluoride-containing mouthrinse, gel, tablets, varnish)?			
For Interviewer:<br>22. [3] Fluoride program (except for water fluoridation)			**0**- Fluoride toothpaste plus constant additional fluoride measures.<br>**1**- Fluoride toothpaste plus inadequate (infrequent) additional fluoride measures.<br>**2**- Fluoride toothpaste only.<br>**3**- No using fluoride toothpaste or other fluoride measures
23. [5] Does your child have his/her own toothbrush?	*Yes*	*No*	If *No*, what is your child's oral care routine?
24. [4] How many times a day do your child's teeth get brushed?			0. None<br>1. Once/day<br>2. 2x/day<br>3. 3x/day<br>4. More than 3x/day
25. [4] How long are your child's teeth brushed each time?			0. Less than 1 min<br>1. 1-2 min<br>2. More than 2 min, less than 3 min<br>3. = 3 mins
26. [4] Who brushes the teeth of your child most frequently?			1. Maid<br>2. Child's parents<br>3. Child himself/herself<br>4. Child's grandparents<br>5. Others _____
27. [5] I don't brush my child's teeth if he/she cries or does not want me too.	*Yes*	*No*	
28. [4] Do you agree with the statement: "I can do a good job brushing my child's teeth each day thoroughly even when I am very busy?"			1. Strongly agree<br>2. Agree<br>3. Neutral<br>4. Disagree<br>5. Totally disagree
29. [1,2,4] Does the child visit the dentist at least once a year?	*Yes*	*No*	
30. [4] If the child does not receive dental check-up every year, what is the main reason?			1. No money<br>2. No time<br>3. Difficult transportation<br>4. Fear of drills, injection, dentists<br>5. His/her teeth do not bother him/her<br>6. Others _______
31. [2] No dental home?<br>episodic dental care?	*Yes*<br>*Yes*	*No*<br>*No*	
32. [4] At what age do you think it's appropriate for your			___ year-old

child to start dental check-ups?			
33. [1,2] Child has developmental problems/special care needs?	*Yes*	*No*	If Yes, ____________________
34. [1,4] How many decayed teeth does your child have in his/her mouth?			0. None<br>1. 1-2 teeth<br>2. 3-4 teeth<br>3. More than 4 teeth<br>4. Don't know
35. [1,2] Plaque is obvious on the child's teeth?	*Yes*	*No*	
and or gums bleed easily?	*Yes*	*No*	
36. [4] Have you ever been told about early childhood caries (tooth decay)?	*Yes*	*No*	If *Yes*, when?_____
37. [4] Do you think baby teeth are important?	*Yes*	*No*	
38. [5] Do you think fixing tooth decay in baby teeth are important?	*Yes*	*No*	If *No*, why?
39. [4] Do you believe that putting baby to bed with a milk bottle is bad for his/her teeth?	*Yes*	*No*	
40. [4] What do you think is the main reason for tooth decay?			1. tooth worms<br>2. heattiness<br>3. ineffective toothbrushing<br>4. sugar<br>5. bacteria
41. [1,2] Child has recent dental restorations?	*Yes*	*No*	
Child information			
42. [1] Child is a recent immigrant (from another area)?	*Yes*	*No*	
43. [1,4] Does your child have any health problem (e.g. allergy, eczema, food allergy, obesity)?	*Yes*	*No*	If *Yes*, what are the problems?
44. [4] Is your child taking medication regularly?	*Yes*	*No*	If *Yes*, what are the medications?
For Interviewer:<br><br>45. [3] Related diseases			**0**- No general diseases of importance related to dental caries<br>**1**- Mild degree disease/conditions indirectly influencing caries process or other conditions which can contribute to higher caries risk, e.g. poor eyesight, inability to move

			**2-** Severe degree, long-lasting disease/conditions. Patient may be bedridden or may need continuous medication for example affecting the saliva secretions
For Interviewer:<br>46. [2] Child has salivary-reducing factors present, including			1. Medications (e.g. some for asthma or hyperactivity)<br>2. Medical (cancer treatment) or genetic factors

[1] CAT

[2] CAMBRA

[3] Cariogram

[4] NUS-CRA

[5] Additional questions
