# Supplementary material for: Prevalence and risk indicators of early childhood caries among toddlers in Caloocan City, Philippines: a cross-sectional study
Source: BMC Oral Health. 2024 May 31;24:642. doi: 10.1186/s12903-024-04407-2 (PMC11141054; doi:10.1186/s12903-024-04407-2)
Supplement: Supplementary file 2 — Supplementary Material 2. [file 12903_2024_4407_MOESM2_ESM.pdf]

PETSA: ___________________________ ID: ___________________

Health Center: _____________________________________

Interview Questionnaire

Mother/Caregiver information			
For Interviewer: (For Question #1, refer to Personal Information Form)<br>1. [1,2]Ano ang katayuan/income bracket sa buhay ng magulang/tagapag-alaga?			1. Low income bracket<br>2. Middle income bracket<br>3. High income bracket
2. [2] Nitong huling 3 buwan, wala ka bang nasirang ngipin?	*Meron*	*Wala*	*Di alam*
3. [1,2] Nagkaroon ka ba ng sirang ngipin sa nakalipas na 12 buwan/1 taon?	*Oo*	*Wala*	*Di alam*
4. [2] Ikaw ba ay ngumunguya ng chewing gum/lozenges 2-4 beses araw-araw? (photo)	*Oo*	*Hindi*	
5. [4] Bilang magulang/tagapag-alaga, nagpapacheck-up ka ba ng ngipin taon-taon?	*Oo*	*Hindi*	
Child feeding practices			
6. [4] Huminto na ba sa pag-breastfeed ang bata? Kung '*Oo*', ilang taong gulang nang huminto ang bata sa pag-breastfeed?	*Oo*	*Hindi*	Kung *Oo*, ___taon ___buwan
7. [3,4] Gaano kadalas kumain (kasama merienda) ang bata sa isang araw? *(flipchart)*			**0-** 0-3 beses<br>**1-** 4-5 beses<br>**2-** 6-7 beses<br>**3-** Higit sa 7 beses kada araw
8. [1,2,4] Gaano kadalas bigyan ng matatamis na merienda o inumin ang bata sa isang araw? (photo) Pakituro dito ang mga madalas i-merienda ng bata. *(flipchart)*			1. Wala<br>2. Minsan<br>3. 2-3 beses<br>4. 4-5 beses<br>5. Higit sa 5 beses kada araw
For Interviewer:<br>9. [3] Content ng pagkain *(Diet content)*			**0** – Maayos na pagkain. Ang asukal at ibang matatamis ay napaka kaunti lang.<br>**1** – Tamang pagkain. Ang asukal at matatamis ay kaunti lang.<br>**2** – Ang pagkain ay laging may matamis.<br>**3** – Hindi tamang pagkain. Mataas ang kasamang asukal at matatamis.
10. [2] Natutulog ba ang bata na may bote sa bibig?	*Oo*	*Wala*	
11. [2] Binibigyan ba ang bata ng dede pag humingi pagkahiga sa gabi?	*Oo*	*Hindi*	

12. [4] Paano matulog ang bata noong 12 buwang gulang siya? *(flipchart)*			1. Walang nakalagay sa bibig<br>2. May dede na laman ay tubig<br>3. May pacifier sa bibig<br>4. Sumususo sa nanay<br>5. May dedeng laman ay gatas/formula/juice<br>6. May laman na matamis sa bibig
13. [4]Nakatanggap ka na ba ng payo tungkol sa relasyon ng pagkain at sirang ngipin mula sa dentista o doktor?	*Oo*	*Hindi*	
Oral health behavior			
14. [4]Nagpatulog ka ba ng bata na pinapadede sa bote sa unang taon niya?			1. Hindi kailanman<br>2. Minsan<br>3. Madalas<br>4. Halos gabi-gabi
15. [1,4]Natutulog ba ang bata nang hindi nagsipilyo matapos kumain ng matamis na merienda?			1. Hindi kailanman<br>2. Minsan<br>3. Madalas<br>4. Halos gabi-gabi
16. [4]Sumasang-ayon ka ba dito: "Nasa akin ang abilidad na pigilan ang madalas na pagmerienda ng matatamis ng bata kahit pa umiyak siya."			1. Matinding pagsang-ayon<br>2. Sang-ayon<br>3. Ayos lang<br>4. Hindi sumasang-ayon<br>5. Matinding di pagsang-ayon
17. [1]Nakakatanggap ba ang bata ng sapat na fluoride sa inumin?	*Oo*	*Hindi*	*Di alam*
o fluoride (vitamin)supplements?	*Oo*	*Hindi*	*Di alam*
18. [2,4]Nakatira ba kayo sa lugar na walang fluoride sa iniinom na tubig?			1. Oo ___taon gulang hanggang ___ taon gulang<br>2. Hindi<br>3. Di sigurado
19. [2]Nakatira ba kayo sa komunidad na may fluoride?	*Oo*	*Hindi*	*Di alam*
o ngumunguya ba siya ng fluoride (vitamin chewable o water-dissolved) supplements?	*Oo*	*Hindi*	*Di alam*
20. [1,2,4]Ang ngipin ng bata ay sinisipilyo gamit ang toothpaste na may fluoride araw-araw?	*Oo*	*Hindi*	*Di alam*
21. [1,4]Ikaw ba o ang dentista ay nakagamit ng produktong may fluoride (fluoride-containing mouthrinse, gel, tablets, varnish) para sa bata?			1. Oo Gaano kadalas? ______<br>2. Hindi<br>3. Di alam
For Interviewer:<br>22. [3]Programa ng fluoride (liban sa water fluoridation) *(Fluoride program)*			**0**- Fluoride toothpaste dagdag lagi ang ibang fluoride measures.<br>**1**- Fluoride toothpaste dagdag ang paminsang fluoride measures.<br>**2**- Fluoride toothpaste lang.

			**3-** Walang fluoride toothpaste o ibang fluoride measures
23. [5] May sarili bang toothbrush ang bata?	*Oo*	*Wala*	Kung *Wala*, ano ang panglinis ng bibig ng bata?
24. [4]Ilang beses sa 1 araw sinisipilyo ang ngipin ng bata?			0. Wala<br>1. Minsan sa 1 araw<br>2. 2 beses sa isang araw<br>3. 3 beses sa isang araw<br>4. Higit 3 beses sa isang araw
25. [4]Gaano katagal sinisipilyo ang ngipin ng bata?			0. Mababa sa 1 minuto<br>1. 1-2 minuto<br>2. Higit sa 2 min, mababa sa 3 min<br>3. Hanggang 3 minuto
26. [4]Sino ang madalas nagsisipilyo sa ngipin ng bata?			1. Kasambahay/Yaya<br>2. Nanay o tatay ng bata<br>3. Ang bata mismo<br>4. Lolo o lola ng bata<br>5. Iba pa _____
27. [5] Sumasang-ayon ka ba dito? "Hindi ko sinisipilyuhan ang bata pag sya ay umiiyak o pag ayaw nya."	*Oo*	*Hindi*	
28. [4]Sumasang-ayon ka ba dito? "Kaya kong sipilyuhin nang maayos ang ngipin ng bata kahit pa busy ako."			1. Matinding pagsang-ayon<br>2. Sang-ayon<br>3. Ayos lang<br>4. Di sang-ayon<br>5. Matinding di pagsang-ayon
29. [1,2,4] Bumibisita ba ang bata sa dentista 1 beses sa 1 taon? *(Kung Oo, proceed to #31.)*	*Oo*	*Hindi*	
30. [4]Kung hindi bumibisita sa dentista ang bata sa 1 taon, ano ang dahilan?			1. Walang pera<br>2. Walang oras<br>3. Mahirap mamasahe<br>4. Takot sa drill, injection, dentista<br>5. Di sumasakit ang ngipin<br>6. Ibang dahilan ________________
31. [2]Ang bata ay walang regular na dentista?<br>o pupunta lang sa dentista pag kailangan?	*Oo*<br>*Oo*	*Hindi*<br>*Hindi*	
32. [4]Sa iyong palagay, sa anong edad dapat unang magpacheck-up ng ngipin ang bata?			_____taon
33. [2]May developmental problems/special care needs ba ang bata?	*Oo*	*Wala*	Kung Oo, ____________________

34. [1,4] Sa palagay mo, ilang sirang ngipin mayroon ang bata ngayon?			0. Wala<br>1. 1-2 ngipin<br>2. 3-4 ngipin<br>3. Higit sa 4 ngipin<br>4. Di alam
35. [1,2]Nakikita mo ba ang dumi sa ngipin ng bata?<br>o madali bang dumugo ang gilagid?	*Oo*<br>*Oo*	*Hindi*<br>*Hindi*	
36. [4]Nasabihan ka na ba tungkol sa early childhood caries (sirang ngipin)?	*Oo*	*Hindi*	Kung *Oo*, kailan yun? ___________
37. [4]Importante ba ang ngipin ng bata?	*Oo*	*Hindi*	
38. [5] Naniniwala ka ba na importante ipagamot ang sirang ngipin ng bata?	*Oo*	*Hindi*	Kung *Hindi*, bakit?
39. [4]Naniniwala ka ba na nakakasama sa ngipin na patulugin ang bata na may dedeng bote sa bibig?	*Oo*	*Hindi*	
40. [4]Ano sa palagay mo ang pangunahing dahilan ng pagkasira ng ngipin?			1. Uod sa ngipin<br>2. Mainit<br>3. Di maayos na pagsisipilyo<br>4. Asukal<br>5. Bacteria
41. [1,2]May bagong pasta ba ang bata?	*Oo*	*Wala*	
Child information			
42. [1]Bagong lipat ba kayo ng bahay mula sa ibang lugar/ immigrant?	*Oo*	*Hindi*	
43. [1,4]May problema ba sa kalusugan ang bata (e.g. allergy, eczema, food allergy, obesity)?	*Oo*	*Wala*	Kung *Oo*, ano ang mga problema?
44. [4] May regular bang iniinom na gamot ang bata?	*Oo*	*Wala*	Kung *Oo*, ano ang mga gamot?
For Interviewer:<br>45. [3]Ano ang pangkalahatang kundisyon ng kalusugan ng bata na maaaring makaapekto sa ngipin at pag-gawa ng laway? *(Related diseases)*			**0**- Walang sakit na maaaring nagdulot ng sirang ngipin.<br>**1**- Mild na sakit/kundisyon na maaaring nagdulot ng sirang ngipin e.g. mahinang paningin, di makagalaw nang maaayos<br>**2**- Malalang sakit/kundisyon. Bata ay nakaratay o may pangangailangan ng tuloy-tuloy na gamot e.g. apektado ang paglalaway.
For Interviewer:<br>46. [2] May kundisyon ba na nagpapakaunti ng laway ng bata, tulad ng			1. Gamot (e.g. asthma o hyperactivity)<br>2. Medical (naggagamot sa cancer) o genetic factors
