# Supplementary material for: Prevalence and risk indicators of early childhood caries among toddlers in Caloocan City, Philippines: a cross-sectional study
Source: BMC Oral Health. 2024 May 31;24:642. doi: 10.1186/s12903-024-04407-2 (PMC11141054; doi:10.1186/s12903-024-04407-2)
Supplement: Supplementary file 3 — Supplementary Material 3. [file 12903_2024_4407_MOESM3_ESM.pdf]

Piliin ang pinaka-angkop na sagot sa mga sumusunod na bilang.

(Choose your most appropriate answer for the following questions.)

7.

- ○ **0-3 beses** **(0-3 meals)**
- ○ **4-5 beses** **(4-5 meals)**
- ○ **6-7 beses** **(6-7 meals)**
- ○ **Higit sa 7 beses kada araw**
  (More than 7 meals per day)

CERTIFIED TRUE COPY OF AN ORIGINAL DOCUMENT
DATE [illegible] MAR 2019

8.

- ○ **Wala** **(None)**
- ○ **Minsan** **(Once)**
- ○ **2-3 beses** **(2-3 times)**
- ○ **4-5 beses** **(4-5 times)**
- ○ **Higit sa 5 beses kada araw**
  (More than 5 times per day)

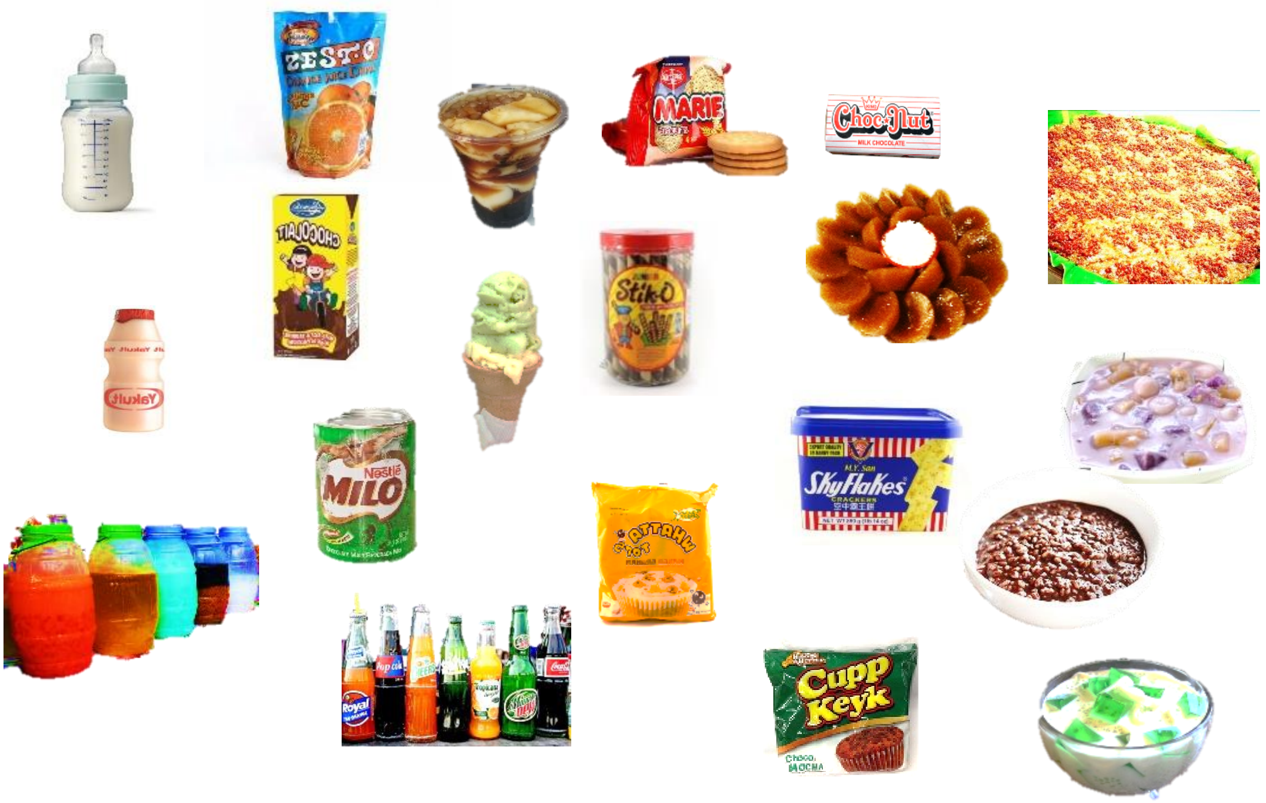

Sourced from Google images. Used for research purposes only. No copyright infringement intended.

12.

- ○ **Walang nakalagay sa bibig** **(Nothing in the mouth)**
- ○ **May dede na laman ay tubig** **(With bottle of water)**
- ○ **May pacifier sa bibig** **(With pacifier only)**
- ○ **Sumususo sa nanay** **(Nursing on mother's breast)**
- ○ **May dedeng laman ay gatas/formula/juice**

  (With bottle of milk/formula/juice)
- ○ **May laman na matamis sa bibig**

  (With something sweet in the mouth)

14.

- ○ **Hindi kailanman** **(Never)**
- ○ **Minsan** **(Occasionally)**
- ○ **Madalas** **(Frequently)**
- ○ **Halos gabi-gabi** **(Almost every night)**

CERTIFIED TRUE COPY OF AN ORIGINAL DOCUMENT
DATE 13 MAR 2019

15.

- **Hindi kailanman** **(Never)**
- **Minsan** **(Occasionally)**
- **Madalas** **(Frequently)**
- **Halos gabi-gabi** **(Almost every night)**

16.

- **Matinding pagsang-ayon** **(Strongly agree)**
- **Sang-ayon** **(Agree)**
- **Ayos lang** **(Neutral)**
- **Hindi sumasang-ayon** **(Disagree)**
- **Matinding di pagsang-ayon**

  (Totally disagree)

17.

CERTIFIED TRUE COPY OF AN ORIGINAL DOCUMENT
CENTER FOR ETHIC IN HUMAN RESEARCH KHON KAEN UNIVERSITY
KKUEC
DATE [illegible] MAR 2019

Fluoride sa inumin: (Fluoride in drinking water:)

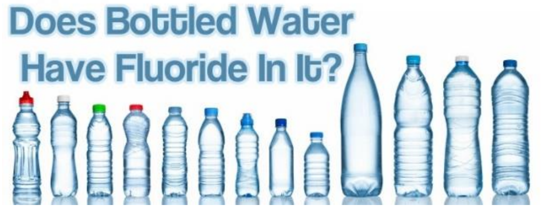

Fluoride (vitamin) supplements:

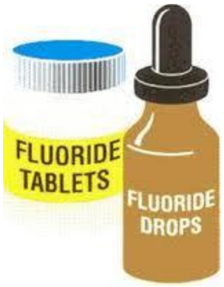

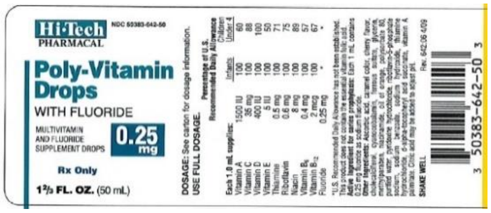

Sourced from Google images. Used for research purposes only. No copyright infringement intended.

18.

- ○ **Oo** **(Yes)**
- ○ **Hindi** **(No)**
- ○ **Di sigurado** **(Not sure)**

20.

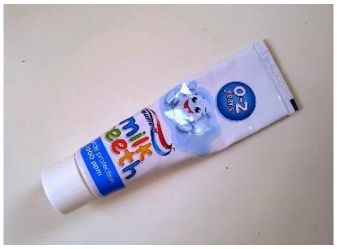

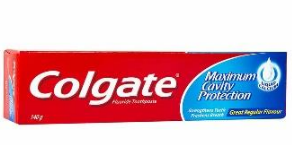

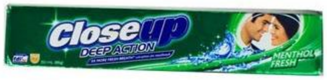

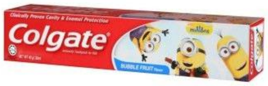

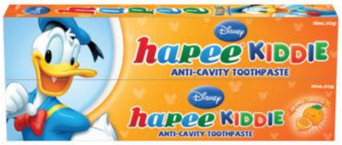

Sourced from Google images. Used for research purposes only. No copyright infringement intended.

21.

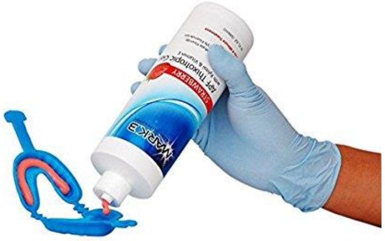

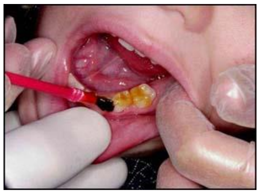

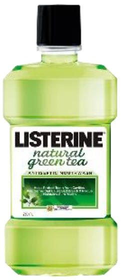

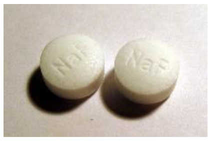

Sourced from Google images. Used for research purposes only. No copyright infringement intended.

CERTIFIED TRUE COPY OF AN ORIGINAL DOCUMENT
DATE 13 MAR 2019

24.

- ○ **Wala (None)**
- ○ **Minsan sa 1 araw (Once/day)**
- ○ **2 beses sa isang araw (2x/day)**
- ○ **3 beses sa isang araw (3x/day)**
- ○ **Higit 3 beses sa isang araw (More than 3x/day)**

25.

- ○ **Mababa sa 1 minuto (Less than 1 minute)**
- ○ **1-2 minuto (1-2 minutes)**
- ○ **Higit sa 2 minuto, mababa sa 3 minuto**

  (More than 2 minutes, less than 3 minutes)
- ○ **Hanggang 3 minuto (=3minutes)**

CERTIFIED TRUE COPY OF AN ORIGINAL DOCUMENT
DATE 12 MAR 2019

26.

- ○ **Kasambahay/Yaya (Maid)**
- ○ **Nanay o tatay ng bata (Child's parents)**
- ○ **Ang bata mismo (Child himself/herself)**
- ○ **Lolo o lola ng bata (Child's grandparents)**
- ○ **Iba pa _____ (Others____)**

28.

- ○ **Matinding pagsang-ayon (Strongly agree)**
- ○ **Sang-ayon (Agree)**
- ○ **Ayos lang (Neutral)**
- ○ **Di sang-ayon Disagree)**
- ○ **Matinding di pagsang-ayon**

  (Totally disagree)

CERTIFIED TRUE COPY OF AN ORIGINAL DOCUMENT
CENTER FOR ETHIC IN HUMAN RESEARCH KHON KAEN UNIVERSITY
DATE 1 2 MAR 2019

30.

- ○ **Walang pera** (No money)
- ○ **Walang oras** (No time)
- ○ **Mahirap mamasahe** (Difficult transportation)
- ○ **Takot sa drill, injection, dentist**

  (Fear of drills, injection, dentists)
- ○ **Di sumasakit ang ngipin**

  (His/her teeth do not bother him/her)
- ○ **Ibang dahilan ________________**

  (Others_____)

34.

- ○ **Wala** (None)
- ○ **1-2 ngipin** (1-2 teeth)
- ○ **3-4 ngipin** (3-4 teeth)
- ○ **Higit sa 4 ngipin** (More than 4 teeth)
- ○ **Di alam** (Don’t know)

CERTIFIED TRUE COPY OF AN ORIGINAL DOCUMENT
DATE [illegible]

40.

- ○ **Uod sa ngipin** **(Tooth worms)**
- ○ **Mainit** **(Heattiness)**
- ○ **Di maayos na pagsisipilyo** **(Ineffective toothbrushing)**
- ○ **Asukal** **(Sugar)**
- ○ **Bacteria**

CERTIFIED TRUE COPY OF AN ORIGINAL DOCUMENT
DATE [illegible] MAR 2019
