# Supplementary material for: Prevalence and risk indicators of early childhood caries among toddlers in Caloocan City, Philippines: a cross-sectional study
Source: BMC Oral Health. 2024 May 31;24:642. doi: 10.1186/s12903-024-04407-2 (PMC11141054; doi:10.1186/s12903-024-04407-2)
Supplement: Supplementary file 4 — Supplementary Material 4. [file 12903_2024_4407_MOESM4_ESM.pdf]

Oral Health Advice

For ______________

FOLLOW-UP DENTAL VISIT AT THE BARANGAY HEALTH CENTER:

____

TOOTH-BRUSHING:

_____ soft bristles; small head toothbrush

_____ duration: 1-2 minutes

_____ clean the tongue

_____ 2 times in a day: (1) morning and (2) last activity before sleeping at night

_____ knee-to-knee position

TOOTHPASTE:

_____ fluoride-containing toothpaste

_____ smear size

_____ pea-sized

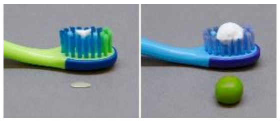

_____ wipe off excess

_____ spit excess, don't rinse

FEEDING HABITS: ____

DENTAL TREATMENT NEED/S:

_____ Prevention: cleaning, topical fluoride application, sealant

_____ Restoration/s on teeth _________________________________

_____ Recall visits: 3 months OR 6 months OR 12 months

_____ Other/s ____

____
Dentist

Date: ________________
