# Supplementary material for: Prevalence and risk indicators of early childhood caries among toddlers in Caloocan City, Philippines: a cross-sectional study
Source: BMC Oral Health. 2024 May 31;24:642. doi: 10.1186/s12903-024-04407-2 (PMC11141054; doi:10.1186/s12903-024-04407-2)
Supplement: Supplementary file 5 — Supplementary Material 5. [file 12903_2024_4407_MOESM5_ESM.pdf]

Oral Health Advice

Para kay _______________

SUSUNOD NA DENTAL CHECK-UP SA BARANGAY HEALTH CENTER:

____

PAGSISIPILYO:

_____ soft bristles; small head na sipilyo

_____ tagal: 1-2 minuto

_____ linisin ang dila

_____ 2 beses sa 1 araw: (1) umaga at (2) huling gawain bago matulog sa gabi

_____ posisyon: tuhod sa tuhod

TOOTHPASTE:

_____ naglalaman ng fluoride (tingnan sa likod ng lalagyan o kahon)

_____ sukat: ga-butil ng bigas

_____ sukat: ga-butil ng mais

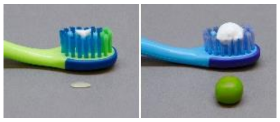

_____ pahiran ang sobrang bula

_____ idura ang sobra, walang mumog

PAGPAPAKAIN SA BATA: ____

PANGANGAILANGANG DENTAL NA PAGGAMOT:

_____ Prevention: cleaning, topical fluoride application, sealant

_____ Pasta sa # ____

_____ Recall visits: 3 months O 6 months O 12 months

_____ Iba pa ____

____

Dentista

Petsa: ________________
